# P-288. From Dialogue to Documentation and Back: A Rule-Based NLP Algorithm to Detect PrEP Discussions and Identify Eligible Patients in the Emergency Department

**DOI:** 10.1093/ofid/ofaf695.509

**Published:** 2026-01-11

**Authors:** Rahma AlDhaheri, Alysse Wurcel

**Affiliations:** Tufts Medical Center, Boston, Massachusetts; Boston Medical Center, Boston, MA

## Abstract

**Background:**

Despite decreasing HIV incidence in the U.S., disparities persist among minoritized populations. Pre-exposure prophylaxis (*PrEP*), a highly effective prevention strategy remains underutilized. Emergency departments (EDs) offer a key opportunity to expand access, especially for individuals facing systemic barriers. While EDs have implemented HIV screening and linkage programs, many *PrEP*-eligible patients remain unrecognized. This highlights the need for scalable tools to identify candidates and prompt *PrEP* discussions. Natural language processing (NLP) can extract relevant information from unstructured electronic health records and has been used to identify clinical concepts. This study aims to develop a rule-based NLP algorithm to detect *PrEP*-related discussions and identify *PrEP*-eligible patients using EHR data.

**Methods:**

This is a retrospective cohort study of ED encounters from April 2022-April 2025. The NLP pipeline is divided into two phases. Phase I: inclusion and exclusion phrase sets were iteratively refined using regular expressions and manual review to enhance classification accuracy. Phase II (currently underway), the algorithm will be applied to a manually annotated dataset. Performance will be evaluated using sensitivity, specificity, and precision. For identifying PrEP-eligible patients, NLP results will be compared to structured data (ICD-10 codes, labs).

**Results:**

Refinement improved the algorithm’s accuracy in detecting PrEP discussions, reducing the number of sentences manually reviewed from 12,016 to 7,255. True positives (TP) increased from 8% in Round 1 to 53.7% in Round 3, while false positives (FP) decreased from 77% to 45.6%; remaining cases were classified as unclear due to insufficient context. Common FP stemmed from unrelated uses of “prep”, for example, “short prep with oral contrast prior to CT” As for PrEP eligibility, 53,175 sentences were initially extracted using 70 phrases, yielding 32.3% TP. In Round 2, refined rules improved performance to 75.6% TP with only 1.2% FP. A representative FP included, “In ambulance she was given IV fentanyl” flagged due to opioid use outside the setting of substance use disorder.

**Conclusion:**

Rule-based NLP shows promise for advancing HIV prevention in the ED.

**Disclosures:**

All Authors: No reported disclosures

